# Promising Extracellular Vesicle-Based Vaccines against Viruses, Including SARS-CoV-2

**DOI:** 10.3390/biology10020094

**Published:** 2021-01-27

**Authors:** Berina Sabanovic, Francesco Piva, Monia Cecati, Matteo Giulietti

**Affiliations:** Department of Specialistic Clinical and Odontostomatological Sciences, Polytechnic University of Marche, 60131 Ancona, Italy; b.sabanovic@pm.univpm.it (B.S.); f.piva@univpm.it (F.P.); m.cecati@univpm.it (M.C.)

**Keywords:** COVID-19, exosomes, vaccine, antigen presentation, antigen display

## Abstract

**Simple Summary:**

Extracellular vesicles (EVs) allow cell-to-cell communication and can induce a strong immune response, by presenting antigens. EVs can be engineered to display viral antigens and so induce high and specific CD8(+) T cell and B cell reactions, highlighting these antigen-presenting EVs as a novel vaccine strategy. EVs present a low basal immunogenic profile and engineered EVs represent a safe, flexible, and efficient strategy for a virus-free vaccine design. Some biotech companies are developing EV-based vaccines against COVID-19, by displaying the SARS-CoV-2 Spike protein on the exosome surface or by delivering mRNAs of viral proteins through EVs.

**Abstract:**

Extracellular vesicles (EVs) are secreted from almost all human cells and mediate intercellular communication by transferring heterogeneous molecules (i.e., DNA, RNAs, proteins, and lipids). In this way, EVs participate in various biological processes, including immune responses. Viruses can hijack EV biogenesis systems for their dissemination, while EVs from infected cells can transfer viral proteins to uninfected cells and to immune cells in order to mask the infection or to trigger a response. Several studies have highlighted the role of native or engineered EVs in the induction of B cell and CD8(+) T cell reactions against viral proteins, strongly suggesting these antigen-presenting EVs as a novel strategy for vaccine design, including the emerging COVID-19. EV-based vaccines overcome some limitations of conventional vaccines and introduce novel unique characteristics useful in vaccine design, including higher bio-safety and efficiency as antigen-presenting systems and as adjuvants. Here, we review the state-of-the-art for antiviral EV-based vaccines, including the ongoing projects of some biotech companies in the development of EV-based vaccines for SARS-CoV-2. Finally, we discuss the limits for further development of this promising class of therapeutic agents.

## 1. Introduction

**Figure 1:**
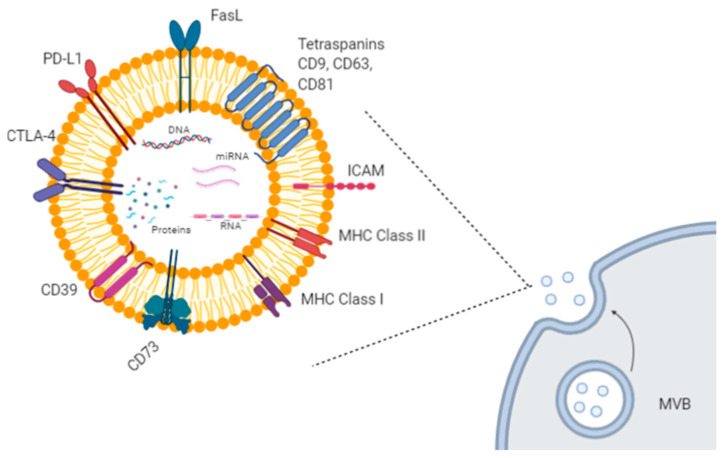
Exosome biogenesis and molecular cargo. Exosomes are extracellular lipid vesicles (EVs) produced within the endosomal compartment called multivesicular bodies (MVB). Exosomes’ cargo includes proteins, DNA, mRNAs, and miRNAs. Some proteins represent exosome markers (e.g., tetraspanins CD63, CD9, CD81), while other proteins are variable depending on the cell type origin, including adhesion molecules (ICAM and integrins), immune-suppressive proteins (CTLA-4, PD-L1, Fas-L, CD39, CD73), major histocompatibility (MHC) molecules, enzymes, and growth factors. Created with BioRender.com.

Extracellular vesicles (EVs) are small lipid particles secreted from almost all human cells types, both healthy and malignant. They can be released either directly from the plasma membrane or upon fusion among multivesicular bodies (MVBs) and the plasma membrane. Based on their size, origin, and cargo heterogeneity (i.e., DNA, proteins, various types of RNAs), EVs have been classified into several groups, such as exosomes, microvesicles, apoptotic bodies, and other vesicle types [1]. Among them, exosomes and microvesicles are very efficient mediators of cell-to-cell communication, by transferring their specific cargo to recipient cells [2,3]. For example, exosomes are involved in the delivery of genetic materials, causing epigenetic modifications in the target cells, in antigen transfer to dendritic cells (DCs) for cross-presentation to T cells, in extracellular matrix remodeling, and in several signaling pathways [2,3] (Figure 1). 

Here, we adhere to the nomenclature guidelines published by the International Society for Extracellular Vesicles (ISEV), which suggests the use of the general term “EVs” instead of “exosomes,” as there is still no definitive distinctive marker of each EV subtype [1].

Viruses and EVs share similar biophysical features due to their small size and similar biochemical composition, which make it difficult to separate them [4]. In addition, many enveloped viruses hijack EV biogenesis mechanisms of infected cells to enhance their dissemination by exploiting the Endosomal Sorting Complex Required for Transport (ESCRT) pathway [5]. For example, the budding of Human Immunodeficiency Virus 1 (HIV-1) at the plasma membrane [6] and the secretion of Hepatitis C Virus (HCV) from host cells require the ESCRT exosomal pathway [7,8], and even rotaviruses and noroviruses have been found within EVs [9]. As some viruses takeover EV biogenesis pathways of infected cells, there has been evolving interest in trying to understand how EV cargo is being altered during viral infections and how its transfer to surrounding uninfected cells could affect viral pathogenesis [10]. For example, HCV glycoproteins [11] and Ebola nucleoproteins [12] have been found in EVs, while an increase in human protein STING (“stimulator of IFN genes”) in CD9+ EVs was observed upon Herpes Simplex-1 (HSV-1) infection [13]. Furthermore, an altered RNA cargo of EVs released from cells infected by Respiratory Syncytial Virus (RSV) resulted in the stimulation of immune responses [14]. Therefore, EVs can both carry viral components to modulate recipient cell susceptibility to infection, and affect the host immune system to mask the infection or to trigger a response [4]. 

Although the viral proteins found in EVs released from infected cells and the hijacked EV biogenesis systems by viruses represent potential therapeutic targets, only few studies have assessed their contribution. Indeed, the main research field remains the use of EVs as delivery systems, as they can be easily loaded with different molecules, including drugs, antibodies, miRNAs, and siRNAs, especially in anti-tumor therapies, resulting in more specific and efficient systems than the carried molecules alone [15]. In particular, EVs showed improved stability, solubility, and biodistribution of loaded therapeutic agents [16]. For example, flotillin+/TSG101+/CD81+ EVs engineered by the addition of monoclonal HIV-1 Env antibodies on the vesicle surface and loaded with the pro-apoptotic miR-143 or the antiretroviral drug curcumin have been successfully used to destroy HIV-1-infected cells [17]. Moreover, EVs accumulate at the site with high vascular permeability, such as tumors, wounds, and sites of inflammation and of infection, due to the enhanced permeability and retention (EPR) effect. At these sites, the vasculature is leakier than healthy blood vessels, resulting in a high deposition of nanoparticles, including EVs for drug-delivery [18]. However, in general, intravenously injected EVs preferentially accumulate in the liver and spleen, probably due to the high levels of macrophages, which take up EVs and participate in the clearance of EVs [19]. In mice, inoculated EVs are rapidly cleared from the circulation, as their half-life was 2–4 min with complete clearance from blood after 4 h [20]. After roughly 30 min, the elimination phase takes over via hepatic and renal clearance, resulting in removal of intravenously injected EVs in a time span from 1 to 6 h [20,21]. Similar clearance levels and biodistribution patterns have also been observed for intravenously injected liposomes [22]. Interestingly, clearance rates and biodistribution profiles are strongly influenced by the administration route of EVs. Indeed, intraperitoneal and subcutaneous injections showed a significantly lower EV accumulation in the liver and spleen [23] and, accordingly, drug-loaded tumor-derived EVs can reach target cells at higher concentrations [22]. Notably, the route of administration is already known to also affect the pattern of metastasis (called organotropism) in mice upon inoculation of human cancer cells [24]. Therefore, clearance, bioavailability and route of administration should be taken into account during the design of both EV-based therapies (e.g., drug-loaded EVs for cancer therapies) and for EV-based vaccines. 

Safety is also a key element for EV-based therapeutics and vaccines. Indeed, EV toxicity and immunogenicity have been assessed by several in vivo studies. EVs released from human mesenchymal stem cells (MSCs) did not induce genotoxic, hematological, or immunological effects during in vitro assays [25]. Human MSC-derived CD81+/CD9+/CD63+ EVs intraperitoneally injected in immunocompetent mice showed no toxicity, even in long-term expositions. Moreover, immunostimulatory effects were not observed, as lymphocyte and myeloid cell profiles and IL-6 and IFN-α levels were not altered [26]. CD81+/CD9+/CD63+ EVs from human embryonic kidney Expi293F cells did not significantly alter the transcriptome of the recipient HepG2 cells and did not induce any signs of hepatotoxicity nor proinflammatory cytokine response in BALB/c mice, after intravenous injection [27]. Unmodified CD63+/TSG101+ EVs from HEK293T cells did not elicit an immune response or toxicity in mice even after repeated intravenous and intraperitoneal administrations for 3 weeks, as observed by blood cell count and blood chemistry panels, histopathological examination, spleen immune cell composition, and evaluations of 23 circulating cytokines [28]. The Codiak BioSciences company (see Section 3 for details about its COVID-19 EV-based vaccine) engineered HEK293-derived EVs to display the anti-tumor cytokine IL-12 (exoIL-12) and, very recently, this company reported that the Phase I trial showed a favorable safety and tolerability profile [29]. It is interesting that the above studies investigated MSC- or HEK293T-derived EVs, as these EVs do not carry class I and class II major histocompatibility (MHC) proteins and B7 co-stimulatory molecules [30,31]. As these molecules are involved in stimulating the immune response, their absence in these allogeneic donor cells ensures the safety of EV vaccines. However, the use of autologous or artificial EVs may be preferable to avoid allogeneic-associated safety issues, but many allogeneic sources already fulfill good manufacturing practices and allow high EV yields, and scaled-up EV production is available. Further studies are needed to definitely ensure EV safety also in clinical settings.

## 2. Antiviral EV-Based Vaccines: General Overview

Due to their involvement in viral infections and the ability of delivering viral antigens to other cells, EVs have also been studied as potential therapeutics in “EV-based vaccine” production. It was already known that CD63+/CD81+ EVs released from monocytes and loaded with viral peptides from influenza virus, Epstein-Barr virus (EBV), and cytomegalovirus (CMV) were able to trigger the release of interferon-γ (IFN-γ) from CD8(+) T cells in an antigen-specific manner [32]. As the IFN-γ induction is a commonly accepted marker to quantify the cellular immune response, such EVs could represent an effective system for vaccine design. In a pivotal study, Montaner-Tarbes et al. demonstrated the presence of porcine respiratory and reproductive syndrome virus (PRRSV) antigens in CD63+/CD81+ EVs from sera of infected pigs [33]. They also proved that viral proteins in EVs were antigenic as immune sera of animals exposed to the PRRSV reacted specifically against EVs isolated from nonviremic pig sera. Notably, this evidence showed that there were viral antigens in circulating EVs although the animals were healed. Recently, the same authors also proved that intramuscular immunizations with their vaccine formulation containing serum EV-enriched fractions from infected pigs were safe, virus-free, and elicited a specific IgG immune response in vaccinated animals. Regarding safety, they showed that even high doses of these EVs did not trigger clinical symptoms associated with PRRSV or with vaccine preparations. Finally, they also demonstrated the feasibility of scaling up the production of these EVs [34]. 

Another approach regarding the design of EV-based vaccines involves the EV engineering, which is the artificial addition of antigens of interest into these vesicles, making them work as antigen-presenting EVs. Two main strategies are possible: The direct modification of EVs after isolation from cells (e.g., by electroporation, bio-conjugation, and click chemistry) or the engineering of the EV donor cells. The latter is the most used method as it allows a continuous production of engineered EVs [35]. By this parental cell-based approach, the proteins of interest can be loaded into the EV lumen or displayed on the EV surface, depending on the particular EV-specific protein used for the fusion with the protein of interest (e.g., Lamp2b, tetraspanins, PDGFR, C1C2 domain of lactadherin for surface display; Ndfip1, ubiquitin tags for lumen loading). RNAs can also be loaded into EVs, by fusing the above-mentioned proteins with RNA-binding proteins such as HuR, TAT, and L7Ae [35].

In this field, Kanuma et al. developed a fused peptide consisting of the antigen ovalbumin and the EV-enriched tetraspanin CD63, in order to produce EVs carrying ovalbumin. Interestingly, they observed that the intradermal immunization of mice with these engineered EVs elicited a specific response by cytotoxic CD8(+) T cells and did not trigger inflammation at the injection site [36]. Similarly, Anticoli et al. used a mutated, and so not immunogenic, HIV Nef protein as an EV-anchoring protein in place of CD63, and fused it with peptides from different viruses, including HPV E7, Ebola VP24, VP40, and NP, Influenza NP, Crimean-Congo Hemorrhagic Fever NP, West Nile NS3, and hepatitis C virus (HCV) NS3. Mouse intramuscular immunizations induced high and specific cytotoxic CD8(+) T cell immunity for all tested viral proteins [37]. This vaccine platform enhanced the low cytotoxic T lymphocyte immunogenicity of EVs, but it still ensured their high biosafety profile. The System Biosciences (SBI) company [38] has developed a EV surface display technology called “XStamp” by employing the C1C2 domain of the peripheral membrane-associated protein lactadherin (or MFGE8) as a further EV-anchoring protein [39]. Recently, EVs from dendritic cells were engineered to present M, NS, and L antigens of Respiratory syncytial virus (RSV). In vitro, they induced both IFNγ production and antigen-specific T cell proliferation. In subcutaneously immunized mice, these EVs elicited antigen-specific CD8+ T cell activation, without side effects in animals [40].

Overall, the findings obtained by serum and engineered EVs undoubtedly suggest the importance of studies on antigen-presenting EVs as a probable novel vaccine strategy. Indeed, current vaccines present several limitations that the implementation of EVs in their production may overcome. Existing antiviral vaccines are based on modified live or attenuated virus, inactivated virus, DNA and RNA vector vaccines, viral subunits, or single peptides. However, some developed vaccines have limited protective immunity, do not elicit long-lasting protection, and may present reversion to virulence, pointing out the need for alternative strategies for vaccine design [41]. 

**Figure 2:**
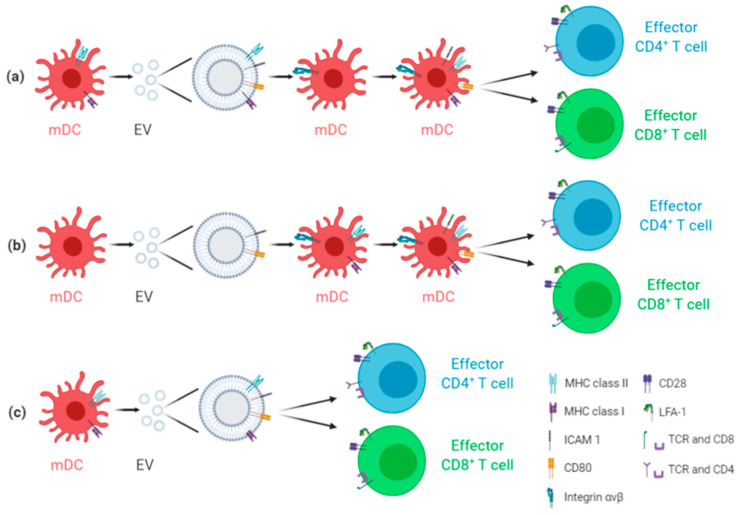
EVs and acquired immunity. EVs from mature dendritic cells (mDCs) can promote T-cell activation through different processes. (**a**) Cross-dressing pattern model: mDCs, once-captured EVs, expose on their surface the antigen-MHC complex promoting the activation of CD4+ or CD8+ T cells. EVs transfer the peptide–MHC complex between different populations of mDCs. (**b**) Cross-presentation pattern model: mDCs expose the EV-delivered antigens on their own MHC complex to activate CD4+ or CD8+ T cells. (**c**) Direct EV-induced T-cell activation model: EVs, released by mDCs, present on their surface the MHC class I and II molecules and directly activate CD4+ or CD8+ T. Created with BioRender.com.

EVs present a low basal immunogenic profile and engineered EVs represent a safe, flexible, and efficient strategy for a virus-free vaccine design. In particular, EVs preserve naïve antigen conformation; they can reach all organs through bodily fluids, especially the sites of infection due to the EPR effect [18]. Moreover, EVs are known to play a role in acquired immunity, as EVs released from macrophages and dendritic cells (DCs) present on their surface the major histocompatibility (MHC) Ι and ΙΙ molecules, B7 co-stimulatory molecules, such as B7.1 (CD80) and B7.2 (CD86), and the adhesion protein ICAM-1 (CD54) [32,42,43]. In particular, the antigen delivery to the T cells can be mediated by EVs through different processes, i.e., cross-dressing pattern, cross-presentation pattern, and direct T-cell activation (Figure 2). In the first model, due to the presence of several adhesion proteins on the EV surface, EVs are efficiently taken up by DCs, which will present to the T cells the entire antigenic peptide–MHC complex of EVs. For the cross-presentation pattern, when DCs capture EVs, they present EV peptides on their own MHC class I and II molecules. Finally, the direct CD4+ or CD8+ T-cell activation can also occur, due to the presence of MHC class I and II molecules on the EV surface [42,43]. 

The study of Montaner-Tarbes et al. also demonstrated that the EV-based vaccine against PRRSV, and not the vaccination with viral peptide alone, was able to induce a high and specific release of IFN-γ, a strong indicator of successful immunization [34]. This result could be explained by the role of EVs in the intercellular communication and antigen presentation. In particular, numerous copies of the same viral protein may be present on the EV surface, facilitating the crosslinking to B-cell receptors. Moreover, viral proteins in EV-based vaccines could indirectly activate B cells and CD8(+) T cells through the antigen cross-presentation process [4,44].

In this regard, a previous seminal study on EV-based vaccines, although not against viruses, demonstrated that CD9+/CD81+ EVs from ovalbumin-pulsed dendritic cells, thus indirectly loaded with ovalbumin, were able to elicit specific T-cell responses in vivo and induce a Th1-type shift, a humoral response (primary and secondary IgG) and the release of IFN-γ [45]. In particular, BALB/c mice were injected intravenously with ovalbumin antigen alone or together with lipopolysaccharides (LPS) or aluminum hydroxide (Alum)—two common vaccine adjuvants—or injected with ovalbumin-loaded EVs. With the exception of ovalbumin alone, strong IgM and IgG primary responses were observed. These tests were repeated on the same mice in order to evaluate the induction of a memory response. The results regarding IgG1 were similar to previous ones, but the production of anti-ovalbumin IgG2a was achieved only in mice immunized with ovalbumin-loaded EVs and not with ovalbumin together with LPS or Alum. It highlights the potential of EVs as immune-modulating adjuvants in the vaccine preparations [45]. Recently, Jesus et al. confirmed the adjuvant activity of EVs in hepatitis B vaccines [46]. In particular, unmodified CD63+/CD81+/Alix+ EVs isolated from LPS-stimulated monocytes induced the release of several cytokines, including IFN-γ, and a shift toward a Th1 response, highlighting an immunomodulatory effect on the cellular immune response in subcutaneously inoculated mice.

Finally, also bacterial EVs, called outer membrane vesicles (OMVs), are arousing increasing interest as they can be exploited as carriers of viral antigens in vaccine design. OMVs are shed from hypervesiculating strains of Gram-negative bacteria (e.g., E. coli) and are noninfectious. For example, OMVs have been engineered to display the highly conserved ectodomain of the M2e protein of influenza virus, suitable for a universal influenza vaccine [47,48]. In mice, engineered M2e-OMVs induced a Th1-biased immune response and antibody-mediated immunity, as strong IgG titers were produced [47]. In order to avoid the lipopolysaccharide (LPS) adverse effects, a genetically modified E. coli strain has been used for the production of only the nonstimulating LPS portion [48].

Overall, engineered EVs are efficient antigen-presenting systems, potent natural adjuvants, and scalable systems with high biosafety, all of which are the characteristics for a good candidate vaccine. Indeed, some biotech companies are currently designing virus-free and adjuvant-free vaccines based on recombinant EVs, such as Ciloa [49], which is developing, in the preclinical phase, candidate vaccines against Chikungunya, Zika, Dengue, and West Nile viruses.

## 3. EV-Based Vaccines: Focus on COVID-19

Since December 2019, we have been faced with the outbreak of the novel coronavirus SARS-CoV-2 (Severe Acute Respiratory Syndrome), and on 11 March 2020, the World Health Organization (WHO) declared pandemic state. The SARS-CoV-2 is a positive singe-stranded RNA virus of the Coronaviridae family, and it shares great similarity with the 2003 SARS-CoV pandemic and the Middle East Respiratory Syndrome (MERS) responsible for the outbreak in 2012 [50]. Some studies have demonstrated that the viral spike glycoprotein (S protein) facilitates the coronavirus infection of the human cells and that, in addition to the S protein, coronaviruses also need two events for cell entry: The receptor binding and the proteolytic cleavage of receptor-bound S protein that makes the spike protein active [50]. Recently, Hoffmann et al. demonstrated that such events are mediated by the human angiotensin-converting enzyme 2 (ACE2) and the Transmembrane Serine Protease 2 (TMPRSS2), respectively [51]. We have recently analyzed the effects of age, sex, diabetes, smoking habits, and pollutant on TMPRSS2 gene expression and their possible involvement in the susceptibility to viral infection and COVID-19 prognosis [52]. 

As the ACE2 receptor is also involved in SARS-CoV cell entry [53], it highlights the high degree of structural homology between S proteins of these Coronaviruses. However, the affinity of the SARS-CoV-2 S protein to the ACE2 is about 10–20 times higher than that of SARS-CoV, partially explaining its higher infectivity and spread [54]. Due to the structural similarity between SARS-CoV and SARS-CoV-2 spike proteins, it is interesting to highlight the results of the development of an EV-based vaccine for SARS-CoV in 2007 [55]. In particular, HSP90+/CD82+ EVs have been engineered in order to strongly enhance the spike protein loading, by creating a chimeric S protein (S^GTM^) as a result of the substitution of its transmembrane domain with that of the G protein of vesicular stomatitis virus (VSV). Interestingly, S^GTM^-containing EVs showed safety and high immunogenicity after footpad injection in mice. In particular, only two injections, instead of several needed for the S protein alone, were sufficient even without any adjuvant for the production of adequate neutralizing antibody titers. Similar results have been obtained with an adenoviral vector expressing S^GTM^, used as the gold standard, thus confirming the efficacy of the EV-based vaccine in inducing an immune response for SARS-CoV. Moreover, this EV-based vaccine induced higher specific antibody titers than those present in SARS patient serum [55]. Overall, this study paved the way for investigation about the EV-based vaccine also against SARS-CoV-2. 

Currently, a specific antiviral treatment for COVID-19 infection is unavailable, but several clinical trials are ongoing about both new therapeutic approaches and vaccines [56,57]. The drug therapies under study include those targeting the virus infection and replication and those targeting the infected host cells and the immune system. Obviously, due to the extreme urgency, clinical trials mainly concern drug repurposing [56,57]. At the moment, different types of vaccines for SARS-CoV-2 are under study and development. Besides the classical approaches of live attenuated vaccines and inactivated virus vaccines, the most common approach is focusing on the viral Spike protein, although with different molecular strategies, such as viral-vector-based vaccines, mRNA vaccines, or those with the full-length S protein or its subunit (receptor binding domain (RBD)) [56,57]. Recently, BNT162b2 (Pfizer, New York City, USA and BioNTech, Mainz, Germany) and mRNA-1273 (Moderna, Cambridge, MA, USA) vaccines have been approved by the medicine regulatory authorities of the UK, USA, and EU, and a mass vaccination campaign started in December, whereas ChAdOx1 nCoV-19 (AstraZeneca, Cambridge, UK and Oxford University, UK) has been very recently approved by UK authority. 

**Figure 3:**
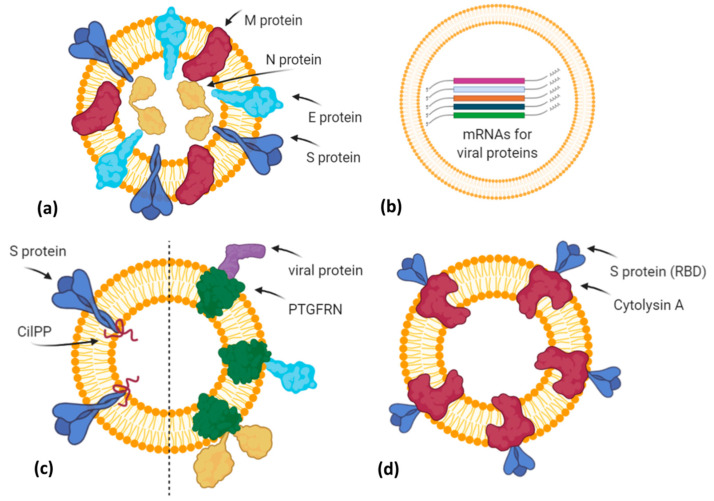
A simplified graphical representation of different EV-based vaccine strategies under development by various biotech companies. (**a**) Capricor Therapeutics transfected HEK293 cells with vectors for Spike, Nucleocapsid, Membrane, and Envelope SARS-CoV-2 proteins in order to release EVs (or virus-like particles) carrying viral antigens in their native conformation; (**b**) Carpicor Therapeutics formulated a COVID-19 vaccine by loading into EVs mRNAs for full-length S protein and for modified S, N, M, and E proteins inserted within Lamp1 protein for a better presentation on MHC I and II molecules. Allele Biotechnology and Pharmaceuticals is also developing an EV-based mRNA vaccine, but details are not available; (**c**) CoVEVax vaccine by Ciloa company consists of EVs displaying full S protein, thanks to the EV-sorting peptide CilPP (left). Similarly, exoVACC™ vaccine of Codiak BioSciences consists of EVs engineered to display still-undeclared SARS-CoV-2 proteins, thanks to the scaffold protein PTGFRN (right); (**d**) bacterial Outer Membrane Vesicles (OMVs) engineered by Versatope Therapeutics for the display of Spike protein (receptor binding domain (RBD) portion) by fusing it with the OMV-anchoring protein cytolysin A (ClyA). Created with BioRender.com.

Additionally, in the last months, vaccines based on EVs or exosome-like vesicles for COVID-19 have been under development by some biotech companies (Figure 3). 

The company Capricor Therapeutics [58] is also working on its EV platform technology as a potential COVID-19 vaccine. Notably, Capricor is developing two different EV-based vaccines designed to stimulate a long-lasting protective immune response to SARS-CoV-2. The first type is an EV display vaccine consisting of human HEK293 cells transfected with vectors for the expression of the four structural SARS-CoV-2 proteins (Spike, Nucleocapsid, Membrane, Envelope). In turn, the released EVs will carry all viral antigens in their native context and conformation (Figure 3a). Previously, it has been demonstrated that immunization with multiple protein forms allows the modulation of the magnitude and the nature of the immune response, in terms of cytokine production and Th1 or Th2 stimulation [59]. However, regarding this vaccine candidate, further details are not available. The second type is an mRNA vaccine formulated by EVs loaded with five different mRNAs coding for modified SARS-CoV-2 Spike, Nucleocapsid, Membrane, and Envelope proteins (LSNME) and for the full-length Spike of Wuhan-1 isolate (S^W1^). In November 2020, Capricor and Johns Hopkins University researchers published, as a pre-print version, encouraging results of pre-clinical trials for their multivalent EV-based mRNA vaccine [60]. They have combined the EV-based mRNA delivery and viral antigen expression compatible for antigen presentation by MHC Class I and II molecules. In particular, they designed mRNAs coding for RBD (receptor binding domain) of the S protein, full-length N protein, and soluble fragments of M and E proteins, but expressed within the extracellular domain of Lamp1 human protein, which is known to be subjected to degradation into short peptides for antigen presentation by the MHC I pathway and, in antigen-presenting cells (APCs), by the MHC II pathway (Figure 3b). HEK293 CD9+/CD63+ EVs loaded with LSNME and functional S^W1^ mRNAs (LSNME/S^W1^ vaccine) were injected intramuscularly into C57BL/6J mice at various concentrations. After the first injection, animals received two further boosters after 3 and 6 weeks, respectively. ELISA assays showed a concentration-dependent antibody response for both N and S proteins, and developed immunity lasting up to 2 months after the second boost injection. Furthermore, the LSNME/S^W1^ vaccine caused a substantial increase in CD4+ and CD8+ T-cells that proliferated upon addition of N and S recombinant proteins to the culture medium of splenocytes, thus confirming that this vaccine formulation is also able to induce cellular immune responses. In particular, S-induced T-cells showed high expression of IFN-γ (Th1 response) but low levels of IL4 (Th2 response). Finally, mice did not show vaccine-induced adverse reactions, such as injection site inflammation, altered body growth, organ morphology, or blood cell profiles. 

The Ciloa company developed a COVID-19 vaccine, called CoVEVax, based on HEK293T-derived CD81+/CD63+/CD9+ EVs. These EVs have been engineered to display the full S protein on their surface, thanks to the fusion with the patented EV-sorting peptide CilPP (Figure 3c). In addition, the S protein has been stabilized by the substitution of two consecutive prolines (K986P, V987P). In a pre-print article, the preclinical results about safety and efficacy have been described [61]. In particular, mice were injected subcutaneously, without adjuvants, with the two components of this vaccine, i.e., the DNA vector for the engineered EVs and the HEK293T-derived engineered EVs. Indeed, only this combination elicited both a humoral and cellular response, measured as levels of specific IgG to S1 or S2 peptide and as antigen-specific IFN-γ production. 

Another biotech company, the Codiak BioSciences [62], in collaboration with the Ragon Institute of MGH, MIT, and Harvard University, is studying the potential of its exoVACC™ vaccine platform for SARS-CoV-2. In particular, exoVACC™ is a modular vaccine system that exploits the unique EV properties, including the simultaneous delivery of specific antigens and immuno-stimulatory adjuvants to the antigen-presenting cells (APCs), for the stimulation of the innate cellular and humoral immune reaction. It is based on the proprietary engEx™ platform for the EV surface display, which utilizes the scaffold protein PTGFRN (Prostaglandin F2 receptor negative regulator), known to preferentially sort in EVs. It is a single-pass transmembrane glycoprotein that enables the high-density surface display of fused proteins of interest, including cytokines, antibody fragments, and other immunomodulatory proteins or specific antigens up to 170 kDa. Due to its high abundance in EVs, PTGFRN showed better packaging and antigen display efficiencies than conventional scaffold proteins, such as CD81, LAMP2B, and the vector system called “pDisplay” based on platelet-derived growth factor receptor (PDGFR) [63]. However, exoVACC™ is still in the research phase, as multiple combinations of SARS-CoV-2 antigens and adjuvants can be produced, and their effectiveness and specificity in vitro and in animal models should be assessed (Figure 3c).

The company Allele Biotechnology and Pharmaceuticals [64] has recently announced the development of an iPSC (induced pluripotent stem cell) line transfected with different mRNAs encoding the SARS-CoV-2 antigen proteins (Figure 3b). These cells can produce a high amount of EVs carrying both viral mRNAs and the corresponding proteins. Allele states that this system overcomes two issues: (i) A vaccine with multiple mRNAs and proteins could have better performances than those with a single mRNA, like Pfizer/BioNTech and Moderna vaccines or those in the ongoing trials; (ii) the Pfizer/BioNTech vaccine needs to be stored at –80 °C, but as EVs protect mRNA from degradation, Allele’s iPSC-derived EVs resulted to be intact for months even when stored at 4 °C. However, similarly to Codiak, no results have been published, nor further methodological details declared.

Finally, Versatope Therapeutics [65] is developing a vaccine against SARS-CoV-2 based on exosome-like nano-sized vesicles called Outer Membrane Vesicles (OMVs). OMVs are lipid vesicles naturally produced by bacteria and present similar characteristics to human EVs. OMVs have been engineered by Versatope in order to display the RBD portion of the Spike protein thanks to its fusion with the OMV-anchoring protein cytolysin A (ClyA), similarly to their candidate vaccine against influenza A virus [47,48] (Figure 3d). In previous studies, OMVs have also been exploited as drug delivery vehicles, cancer immunotherapy agents, immune adjuvants, and vaccines against their parent bacteria [66] or engineered to express antigens of interest from influenza A H1N1 Virus and MERS-CoV [67], in order to elicit protection mediated by antigen cross-presentation to CD8(+) T cells [68]. The concerns about OMV biosafety, due to their bacterial origin and consequent possible inflammatory response, seem to be solved by chemical and genetic approaches that efficiently reduce OMV reactogenicity in humans. In particular, the use of nonionic detergents and chelating agents causes the reduction or dissolvement of OMV lipopolysaccharides (LPS) after the isolation of these bacterial vesicles, whereas the genetic engineering of genes involved in the biosynthetic pathway of LPS allows for the production of recombinant OMVs with attenuated LPS forms [69].

In case these EV-based vaccines demonstrate lower immune responses, it could be useful to consider the use of the modified mRNA coding for the prefusion conformation of the Spike protein, as employed so far by Moderna and Pfizer/BioNTech. This modified mRNA could be loaded directly into extracellular vesicles or into cells by transfection in order to produce vesicles carrying this mRNA. The Spike protein undergoes structural rearrangements in order to fuse with the host cell membrane, from an unstable prefusion conformation to a highly stable post-fusion conformation. It has been demonstrated that stabilization of prefusion-immunogens, preserving epitopes subjected to neutralization, is a promising vaccine strategy for enveloped viruses [70]. Previously, it has been described as a successful stabilization of MERS-CoV and SARS-CoV in prefusion conformation by 2 proline substitutions (2P) in the central helix and heptad repeat 1 of the Spike protein [71]. This mutation is responsible for higher immunogenicity of the MERS-CoV S(2P) protein than wild-type protein, and better stability of the S protein of other betacoronaviruses. This finding indicated a possible strategy for development of vaccines against betacoronaviruses, including SARS-CoV-2. Indeed, the company Moderna substituted the two prolines in SARS-CoV-2 S protein residues 986 and 987 in order to obtain prefusion-stabilized protein and shortly after started the production of mRNA-LNP for SARS-CoV-2 S(2P) protein (mRNA-1273) [70]. Similarly, Pfizer/BioNTech also based their vaccines on mRNA coding for S protein (2P), but with additional modifications. In particular, their vaccine is formulated as a lipid-nanoparticle loaded with N-methyl-pseudouridine (m1Ψ) nucleoside-modified mRNA coding for S protein (2P) containing a native furin cleavage site resulting in two cleavage fragments. The methyl-pseudouridine modification and the optimization of noncoding elements are responsible for enhanced in vitro RNA translation and reduced immune sensing [72]. Previously, m1Ψ-modified mRNA vaccines have been found immunogenic for other viruses, including Zika and HIV-1 [73,74]. Finally, as this recombinant trimeric S (2P) protein is still able to bind the human ACE2 receptor and human anti-RBD antibodies with high affinity, it proves its structural and functional integrity [72].

## 4. Conclusions

In the recent years, EVs have become a focus of many scientific studies, whose results have indicated many possibilities of their use as biomarkers, therapeutics, and more recently, also as vaccines. Interestingly, in a recent clinical trial, the treatment of COVID-19 patients with EVs from allogenic bone marrow mesenchymal stem cells showed promising results, probably through the downregulation of the cytokine storm [75]. As for the EV-based vaccines, their first applications have been in the field of oncology, and only recently have there been some developments in the anti-viral therapies, both in industry and research settings. The current SARS-CoV-2 pandemics could additionally push forward EV studies, especially on the development of EV vaccines. As discussed above, there are numerous advantages of EV-based vaccines than the conventional ones; however, there are still some issues that need to be faced for rapid clinical application. In particular, the phases of the selection and validation of the optimal combination of antigens and adjuvants are very time-consuming. Additionally, this composition should not include other immunogenic antigens (i.e., deriving from in vitro cell line systems used for the EV production) besides the desired ones. It should also be assessed the eventual undesirable induction of further molecular responses in the EV recipient cells that could reduce the vaccine efficacy. Finally, the reproducibility of the identified vaccine composition must be guaranteed. Despite these points, recent data and the substantial investments by biotech companies lead us to suppose that efficient antiviral EV-based vaccines will be available in the near future. However, although humoral and cellular responses were observed in all studies, protective immunity has not yet been evaluated, so protection from future infections remains an open question.

## Data Availability

Not applicable.
